# Maternal exposure to intimate partner violence and its association with diarrhoea prevalence and treatment among children under five in Nepal

**DOI:** 10.1186/s12889-026-26825-3

**Published:** 2026-03-03

**Authors:** Divya Lakhotia, Mariano Salazar

**Affiliations:** 1 International Health Policy Program Foundation, Nonthaburi, Thailand; 2 Department of Global Public Health, Karolinska Institutet, Stockholm, Sweden

**Keywords:** Intimate partner violence, Child diarrhoea, Treatment-seeking behaviour, DHS

## Abstract

**Background:**

Diarrhoeal disease remains a critical public health concern, and a leading cause of death among children under the age of five. Increasingly, studies are also showing a relationship between child diarrhoea and maternal exposure to intimate partner violence (IPV). This study assessed the relationship between maternal experience of different forms of IPV and child diarrhoea prevalence and treatment-seeking in Nepal.

**Methods:**

Study participants included children under five years whose mothers were asked about IPV exposure in the 2022 Nepal Demographic and Health Survey (*n* = 1,959). The main outcomes were: (1) Diarrhoea prevalence in the last two weeks, and (2) Diarrhoea treatment. Main exposures assessed were last twelve months maternal experience of physical, sexual, emotional IPV, and controlling behaviours. Multivariable Poisson regression with robust standard errors were used to estimate adjusted prevalence ratios and 95% CI.

**Results:**

Diarrhoea prevalence was 8.9%, with treatment sought for 56.6% of cases. Children whose mothers were exposed to sexual IPV (aPR: 1.94 95% CI: 1.17–3.21), emotional IPV (aPR: 1.87; 95% CI: 1.17-3.00), controlling behaviour (aPR: 1.64; 95% CI: 1.14–2.35), or any form of IPV (aPR: 1.71; 95% CI: 1.19–2.45) had higher diarrhoea prevalence in the last 2 weeks. Maternal exposure to emotional IPV (aPR: 0.32; 95% CI: 0.15–0.70) was associated with lower prevalence of seeking treatment. In sex-stratified analyses, male children whose mothers experienced emotional IPV (aPR: 1.91; 95% CI: 1.01–3.63) or controlling behaviour (aPR: 1.68; 95% CI: 1.08–2.62), and female children whose mothers experienced any form of IPV (aPR: 1.68; 95% CI: 1.06–2.66) had significantly higher diarrhoea prevalence. Conversely, maternal exposure to emotional IPV was associated with significantly lower treatment-seeking among female children only (aPR: 0.09; 95% CI: 0.02–0.35).

**Conclusion:**

Maternal exposure to IPV was associated with a higher prevalence of diarrhoea and lower treatment-seeking behaviour, with key differences by child sex, underscoring the need for gender-sensitive, integrated maternal and child health interventions that address IPV.

## Background

Diarrhoeal disease remains a critical public health concern, and a leading cause of death among children under the age of five around the world [1]. Globally, there are an estimated 1.7 billion cases of childhood diarrhoea annually, making it one of the primary contributors to child malnutrition and mortality [2]. In 2021, the global diarrhoea mortality rate among male children under five was 54.5 per 100,000 population, compared to 48.7 among females. In South Asia, this rate was 37.1 for male and 33.6 for female children [3]. Although significant improvements have been made in reducing under-five mortality, from 9.9 million deaths in 2000 to 5 million in 2020, diarrhoea continues to claim a substantial number of lives, particularly in low- and middle-income countries (LMICs) [4].

Diarrhoea-related morbidity remains high even as mortality rates decline, placing a heavy economic and social burden on affected communities. The youngest children, especially those in marginalized or resource-limited settings, are particularly susceptible to severe illness. Healthcare-seeking behaviour plays a critical role in determining outcomes of childhood illnesses. Timely and appropriate treatment, often driven by maternal decisions, is vital to reducing morbidity and mortality [5]. The World Health Organization (WHO) estimates that appropriate care-seeking could prevent up to 20% of child deaths [6]. Yet, barriers to access and utilization of healthcare services continue to exist.

Known risk-factors for diarrhoea among children under the age of five include low birthweight and short gestation, unsafe water and sanitation, and suboptimal breastfeeding [3]. Increasingly, studies are also showing a relationship between child diarrhoea and maternal exposure to intimate partner violence (IPV) [7–9]. IPV is a prevalent public health issue that affects three in ten women globally [10]. IPV includes physical, sexual, emotional violence, and controlling behaviours by a current or former partner [11] and carries severe consequences for women’s health, autonomy, and safety. The negative health and social outcomes of IPV extend beyond women themselves, influencing family dynamics and perpetuating cycles of disadvantage across generations [12]. In South Asia, the prevalence of IPV exceeds the global average by 35%, driven by entrenched patriarchal norms and socio-cultural inequalities. These structural factors limit women’s access to education, employment, and decision-making power, reinforcing their vulnerability to violence [13]. There is a global recognition on the urgency of eliminating violence against women and girls (VAWG), with Sustainable Development Goal (SDG) 5.2 specifically calling for the elimination of all forms of VAWG [14].

Maternal exposure to IPV is also harmful for their children. A study published in 2011 shows a conceptual pathway linking children’s exposure to domestic violence (CEDV) to adverse health outcomes. It shows how CEDV results in direct and indirect harm; ranging from prenatal exposure to violence-induced stress, to disruption in caregiving and healthcare-seeking behaviour [15]. Children of mothers who experience IPV are more likely to face developmental delays, poorer physical and mental health [16–19], which translates into lower quality of life (HRQOL) [20]. Studies have shown that children of women who experienced physical, sexual or emotional IPV have higher risk of being low-birth weight [21], being stunted [22], reporting having diarrhoea [8, 9, 23], and acute respiratory infections (ARIs) or fever in the last two weeks [24, 25]. Children can also be indirectly affected by their mothers´ exposure to violence [15]. Maternal care can be disrupted as women struggle with the stress and poor physical and/or mental health emanating from dealing with a violent partner. This can limit mother´s engagement with health services, which can negatively impair child health [15]. These factors can compromise a child’s physiological development and increase their vulnerability to illness.

Nepal has made commendable progress in reducing child mortality, achieving its Millennium Development Goal by 2015 [26]. However, challenges in reducing child health mortality and morbidity remain pressing. As of 2022, Nepal’s under-five mortality rate stands at 27.3 deaths per 1,000 live births [27]. Diarrhoea remains one of the leading causes of illness and death among children under five in Nepal, and contributes to long-term consequences such as stunting and malnutrition [28]. Diarrhoeal disease is particularly sensitive to caregiving practices, household resources, and timely healthcare-seeking [29], all of which may be adversely affected by maternal exposure to IPV. It is also a widely used and consistently measured child morbidity outcome in population-based surveys, making it a robust indicator for examining downstream child health outcomes associated with maternal exposure to IPV at the population level.

According to the 2016 Nepal Demographic and Health Survey (DHS), the prevalence of diarrhoea in the two weeks preceding the survey among children under five varied geographically, ranging from 3.7% to 9.0% across provinces [28]. Emerging evidence also shows that Nepal’s vulnerability to climate change may worsen the problem; even minor increases in temperature and rainfall have been associated with rises in diarrhoea incidence [30].

While previous studies have reported an association between maternal exposure to IPV and increased risk of childhood diarrhoea [8, 9, 23], many are limited in scope, often focusing only on physical or sexual violence. Emotional violence and controlling behaviour are the most common forms of IPV [31, 32] and have also been associated with poor mental health among women [33], unintended pregnancies [34], and children´s impaired long-term growth [35, 36]. Therefore, it is important to examine whether, these often-understudied forms of IPV, are associated with diarrhoea prevalence and treatment seeking among children in this setting. This study contributes to the existing literature by analysing a nationally representative dataset from Nepal to examine associations between multiple forms of IPV—including emotional IPV and controlling behaviours—and child diarrhoea outcomes. Importantly, it also explores whether these associations differ for male and female children, offering new insights into the gendered relationship of IPV on child health.

## Method

### Study design and sample

**Fig. 1:**
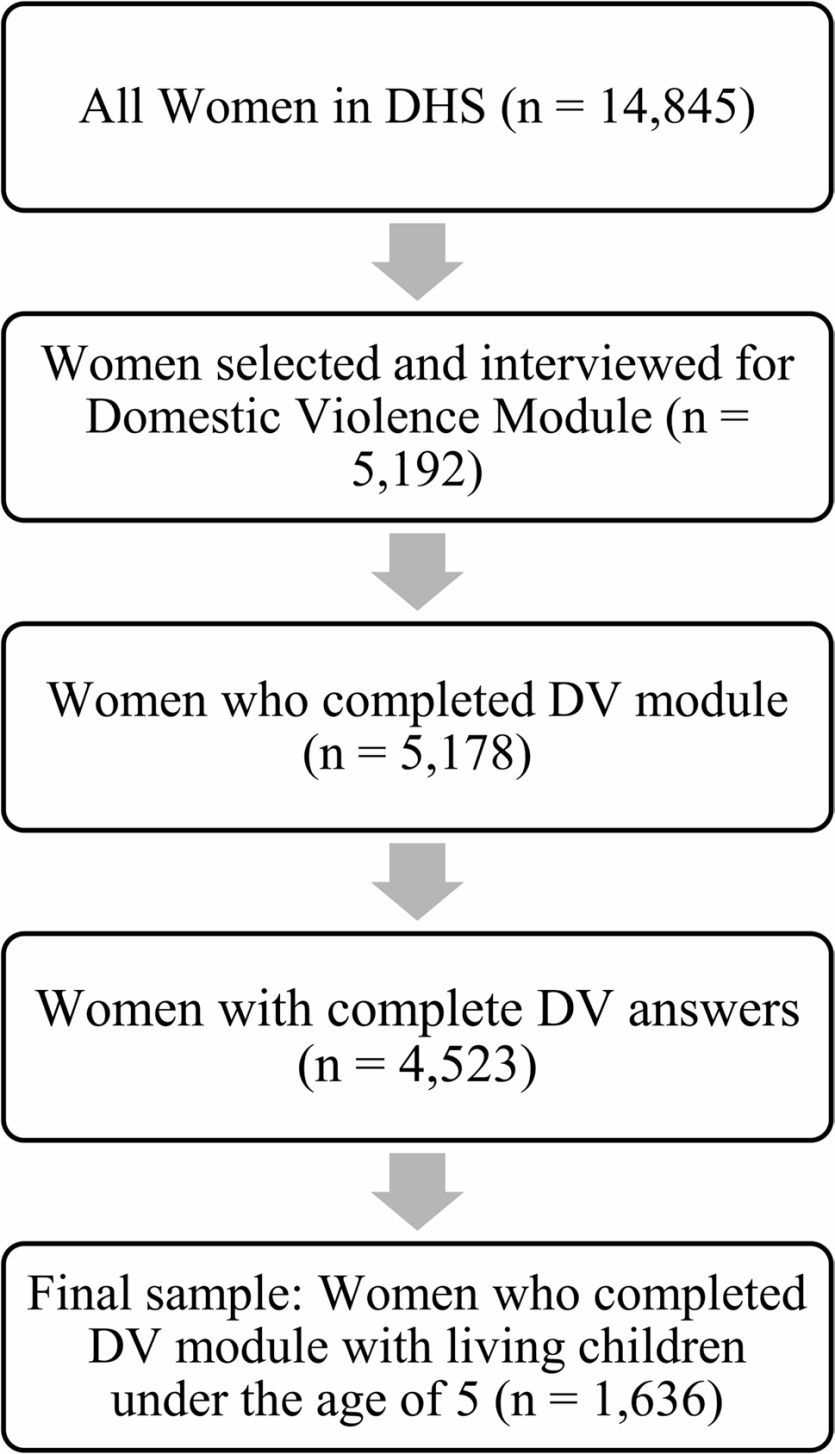
Flowchart showing study sample size and selection

This study is a cross-sectional analysis of data from the 2022 Nepal Demographic and Health Survey (DHS), which is a nationally representative household survey [37]. Among the 14,845 women surveyed for the DHS, only 5,192 women were selected for the domestic violence module (Fig. 1). The module was administered to a subsample of households that were initially selected for the men’s survey. Within these households, only one eligible woman aged 15–49 was randomly selected to participate in the domestic violence module.

Of the 5,192 women selected, 5,178 completed the module. Women with missing data were excluded, leaving a total of 4,523 with complete responses to the questions relevant to this study. Among them, 1,636 had at least one child under the age of five, forming the final study sample: 1,636 women and 1,959 children under five (Fig. 1).

### Study variables

#### Outcome

Two binary outcome variables were used: 1) whether the child had diarrhoea in the two weeks preceding the survey, and 2) among children with diarrhoea, whether care or treatment was sought from any provider. The 2022 Nepal DHS measured diarrhoea prevalence in children under age 5 by asking mothers or caregivers if the child had diarrhoea in the last 24 h or within the last two weeks preceding the survey. A binary variable was created to assess whether a child had diarrhoea within the last two weeks preceding the survey. Diarrhoea treatment seeking was measured by asking mothers or caregivers whether they sought advice or treatment for their child’s diarrhoea if the child had experienced it in the 2 weeks preceding the survey. Seeking advice or treatment could involve going to various sources, including public sector facilities such as government hospitals, health centres, and health posts; private medical sector facilities such as private hospitals, private clinics, and pharmacies; other sources like NGOs, shops, and traditional practitioners. A binary variable was used to assess whether a child with diarrhoea had received treatment or not.

#### Main exposures

The primary exposure was maternal experience of IPV within the 12 months preceding the survey. IPV was assessed among women based on responses to a standardised module in the DHS, covering four forms of violence: physical, sexual, emotional, and controlling behaviours. Binary variables were created for each type of IPV to indicate whether the woman had experienced that form of violence in the past year.

Physical violence included pushing, shaking, throwing something at them, slapping, twisting their arm, or pulling their hair, as well as more severe acts such as punching them with a fist or an object, kicking, dragging, or beating them up, attempting to choke or burn them on purpose, or attacking them with a knife, gun, or other weapon. Sexual violence included being physically forced to have sexual intercourse or engage in unwanted sexual acts. Emotional violence was defined as being humiliated, threatened, or insulted in a way that made the respondent feel bad about herself. Controlling behaviours included the partner displaying jealousy or anger when the respondent talks to other men, accusations of infidelity, refusal to permit the respondent to meet her female friends, attempts to limit the respondent’s contact with her family, or insistence on knowing the respondent’s whereabouts at all times. A composite binary variable, any IPV, was also created to capture whether the woman experienced at least one of the four forms of violence in the past 12 months.

#### Covariates

Covariates included province (Madhesh, Koshi, Bagmati, Gandaki, Lumbini, Karnali, and Sudurpashchim), urban/rural residence, religion (Hindu, Buddhist, Muslim, Kirat, Christian, and other), ethnicity (Brahmin/Chhetri, Dalit, Janajati, Muslim, Newar, and other), maternal and partner education (no education, basic, secondary, and higher), maternal working status (working or not working), maternal age group, child’s sex (male or female), child’s age in months, birth order, and number of children under five in the household. Poverty was measured using the DHS household wealth index (poorest, poorer, middle, richer, and richest). This composite measure is derived through principal component analysis and incorporates variables such as type of flooring, water supply, access to and type of sanitation facilities, and the availability of electricity, television, and radio in the household, among other assets [38].

### Statistical Analysis

Data cleaning and statistical analysis were conducted in R Studio. Percentages, medians, and interquartile range (IQR) were used to summarize the data. Medians and IQR were used since the standard errors of the continuous variables were not normally distributed (Kolmogorov-Smirnov Test p value < 0.05). Multivariable Poisson regression models with robust standard errors [39–42] was used to estimate prevalence ratios (PRs) measuring the association between IPV and the outcome variables adjusting for relevant covariates. This method was chosen because logistic regression has been shown to overestimate the associations between variables in cross-sectional studies [40, 41] and because “*the PR ratio is more interpretable and easier to communicate than the odds ratio*” [40].

Each IPV variable was modelled separately. Covariates included in the model were selected based on previous studies [25, 43, 44] and theoretical understanding of the relationship between exposure and outcome. These co-variables were country region, residence, religion, household wealth index, partner’s education level, mother’s age, mother’s education level, mother’s occupation, number of children under five years in the household, child’s birth order number, child’s age in months, and child’s sex.

The analysis accounted for the DHS’s complex sampling design in accordance with DHS Program guidelines [45], specifying the primary sampling units (clusters), the stratification variable, and the domestic violence weight, as maternal experience of IPV was the main exposure. Additional stratified analyses were conducted by sex of the child. Statistical significance was set at *p* < 0.05. Multicollinearity was assessed using variable inflation factor (VIF). Multicollinearity was present if individual variable VIF was 5 or more. Maternal union status and number of children ever born were excluded from the final models due to multicollinearity. The final overall model VIF was 1.41 with remaining co-variables VIFs ranging from 1.23 to 1.64.

## Results

The weighted diarrhoea prevalence was 8.9%. Of these, 56.6% children received treatment for diarrhoea. Bivariate analysis showed that diarrhoea occurrence was associated with household poverty level, lower partner’s education, and child’s younger age (Table 1, *p* < 0.05). Regarding treatment, first born children and those whose mother’s religion was Hindu received more treatment than those who were not (Table 1, *p* < 0.05).

**Table 1:** Bivariate analysis of factors associated with diarrhoea prevalence and diarrhoea treatment; unweighted column percentages, medians, interquartile ranges (IQR), and p values are shown

Variable	Diarrhoea Yes (*N* = 177)	Diarrhoea No (*N* = 1,782)	*p*-value	Treatment Yes (*N* = 100)	Treatment No (*N* = 77)	*p*-value
Region			0.24			0.15
Madhesh	39 (22%)	360 (20%)		19 (19%)	20 (26%)	
Koshi	28 (16%)	248 (14%)		13 (13%)	15 (20%)	
Bagmati	28 (16%)	218 (12%)		18 (18%)	10 (13%)	
Gandaki	9 (5%)	142 (8%)		2 (2%)	7 (9%)	
Lumbini	17 (10%)	272 (15%)		11 (11%)	6 (8%)	
Karnali	32 (18%)	290 (16%)		21 (21%)	11 (14%)	
Sudurpashchim	24 (14%)	252 (14%)		16 (16%)	8 (10%)	
Residence (Rural)	81 (46%)	942 (53%)	0.08	46 (46%)	35 (46%)	1.00
Mother’s Religion			0.16			**0.03**
Hindu	146 (83%)	1520 (85%)		86 (86%)	60 (78%)	
Buddhist	6 (3%)	104 (6%)		1 (1%)	5 (7%)	
Muslim	8 (5%)	60 (3%)		3 (3%)	5 (7%)	
Kirat	9 (5%)	46 (3%)		3 (3%)	6 (8%)	
Christian	8 (5%)	50 (3%)		7 (7%)	1 (1%)	
Other	0 (0%)	2 (0%)		0 (0%)	0 (0%)	
Ethnicity			0.27			0.14
Brahmin/Chhetri	45 (25%)	603 (34%)		30 (30%)	15 (20%)	
Dalit	44 (25%)	355 (20%)		29 (29%)	15 (20%)	
Janajati	49 (28%)	490 (28%)		25 (25%)	24 (31%)	
Muslim	8 (5%)	64 (4%)		3 (3%)	5 (7%)	
Newar	3 (2%)	31 (2%)		1 (1%)	2 (3%)	
Other	28 (16%)	239 (13%)		12 (12%)	16 (21%)	
Household Wealth Index			**0.04**			0.14
Poorest	56 (32%)	683 (38%)		35 (35%)	21 (27%)	
Poorer	42 (24%)	363 (20%)		27 (27%)	15 (20%)	
Middle	44 (25%)	302 (17%)		23 (23%)	21 (27%)	
Richer	19 (11%)	260 (15%)		6 (6%)	13 (17%)	
Richest	16 (9%)	174 (10%)		9 (9%)	7 (9%)	
Partner’s Education Level			**0.01**			0.78
No Education	21 (12%)	199 (11%)		12 (12%)	9 (12%)	
Basic	87 (51%)	694 (40%)		47 (49%)	40 (53%)	
Secondary	59 (34%)	735 (42%)		34 (35%)	25 (33%)	
Higher	5 (3%)	119 (7%)		4 (4%)	1 (1%)	
Mother’s Age Group			0.53			0.41
15–19	13 (7%)	91 (5%)		10 (10%)	3 (4%)	
20–24	56 (32%)	550 (31%)		32 (32%)	24 (31%)	
25–29	57 (32%)	650 (37%)		33 (33%)	24 (31%)	
30–34	30 (17%)	329 (19%)		15 (15%)	15 (20%)	
35–39	18 (10%)	123 (7%)		9 (9%)	9 (12%)	
40–44	2 (1%)	25 (1%)		0 (0%)	2 (3%)	
45–49	1 (1%)	14 (1%)		1 (1%)	0 (0%)	
Mother’s Education Level			0.05			0.91
No Education	49 (28%)	396 (22%)		27 (27%)	22 (29%)	
Basic	71 (40%)	619 (35%)		41 (41%)	30 (39%)	
Secondary	53 (30%)	708 (40%)		29 (29%)	24 (31%)	
Higher	4 (2%)	59 (3%)		3 (3%)	1 (1%)	
Mother’s Working Status (Not Working Ever or Since 12 Months)	50 (28%)	463 (26%)	0.57	25 (25%)	25 (33%)	0.31
Child Sex (Male)	99 (56%)	948 (53%)	0.54	52 (52%)	47 (61%)	0.29
Child Birth Order			0.86			**0.03**
1	64 (36%)	664 (37%)		44 (44%)	20 (26%)	
2	66 (37%)	615 (35%)		31 (31%)	35 (46%)	
3	27 (15%)	272 (15%)		17 (17%)	10 (13%)	
4+	20 (11%)	231 (13%)		8 (8%)	12 (16%)	
Number of Children in the Household < 5 years. Median (IQR).	1 [1–2]	1 [1–2]	0.39	1 [1–2]	1 [1–2]	0.99
Child Age in Months Median (IQR).	24 [10–38]	32 [18–46]	**0.00**	27 [13–40]	23 [12–35]	0.30

Results of the categorical variables are expressed as frequency (percentage), continuous variables are expressed as median and interquartile range (IQR)

Bivariate analysis of maternal exposure to IPV and child diarrhoea prevalence and diarrhoea treatment showed significant associations between sexual violence, controlling behaviours, and any form of IPV and child diarrhoea prevalence (Table 2, *p* < 0.05). Regarding treatment-seeking, maternal exposure to emotional IPV was significantly associated with lower treatment seeking for their children with diarrhoea (Table 2, *p* < 0.05).

**Table 2:** Bivariate analysis of maternal exposure to IPV and child diarrhoea prevalence and diarrhoea treatment; weighted column percentages and p-values are shown

Variable	Child Diarrhoea Yes	Child Diarrhoea No	*p*-value	Treatment for Diarrhoea Yes	Treatment for Diarrhoea No	*p*-value
Physical Violence	17%	16%	0.72	12%	23%	0.12
Sexual Violence	8%	4%	**0.04**	8%	16%	0.05
Emotional Violence	19%	12%	0.07	4%	12%	**0.04**
Controlling Behaviour	44%	32%	**0.01**	12%	34%	0.73
Any Violence	54%	39%	**0.01**	11%	31%	0.29

Results are expressed as percentage of women who experienced IPV

After adjusting for covariates, several forms of IPV were significantly associated with increased diarrhoea prevalence (Table 3). Sexual violence had the strongest association (aPR: 1.94; 95% CI: 1.17–3.21), indicating that the prevalence of diarrhoea was 94% higher among children whose mothers experienced sexual violence in the past 12 months compared to those who did not. Emotional violence (aPR: 1.87; 95% CI: 1.17-3.00) was associated with an 87% higher prevalence of diarrhoea. Controlling behaviours (aPR: 1.64; 95% CI: 1.14–2.35) and any violence (aPR: 1.71; 95% CI: 1.19–2.45) were associated with 64% and 71% higher prevalence of diarrhoea, respectively.

**Table 3:** Adjusted PR and 95% CI of the association between maternal exposure to different forms of IPV and child’s diarrhoea prevalence in the two weeks preceding the survey, stratified by the sex of the child

Violence last 12 months	aPR^1^ (95% CI) Overall (*N* = 1,959)	aPR^2^ (95% CI) Male (*N* = 1,047)	aPR^2^ (95% CI) Female (*N* = 912)
Physical	1.09 (0.62–1.92)	0.93 (0.46–1.90)	1.33 (0.60–2.95)
Sexual	**1.94 (1.17–3.21)**	1.75 (0.78–3.92)	1.96 (0.92–4.15)
Emotional	**1.87 (1.17–3.00)**	**1.91 (1.01–3.63)**	1.44 (0.64–3.25)
Controlling behaviour	**1.64 (1.14–2.35)**	**1.68 (1.08–2.62)**	1.45 (0.90–2.35)
Any violence	**1.71 (1.19–2.45)**	1.55 (0.91–2.63)	**1.68 (1.06–2.66)**

aPR^1^ = Prevalence Ratio adjusted for covariates including region, residence, religion, household wealth index, partner’s education level, mother’s age, mother's education level, mother's occupation, number of children under five years in the household, child’s birth order number, child’s age in months, and child’s sex

aPR^2^ = Prevalence Ratio adjusted for covariates including region, residence, religion, household wealth index, partner’s education level, mother’s age, mother's education level, mother's occupation, number of children under five years in the household, child’s birth order number, and child’s age in months

*CI* Confidence Interval

Sex-stratified analysis revealed notable differences in these associations. Among male children, maternal exposure to emotional violence (aPR: 1.91; 95% CI: 1.01–3.63) was associated with a 91% higher prevalence of diarrhoea. Controlling behaviour (aPR: 1.68; 95% CI: 1.08–2.62) was associated with a 68% higher prevalence of diarrhoea. In contrast, maternal exposure to any violence (aPR: 1.68; 95% CI: 1.06–2.66) was associated with a 68% higher prevalence of diarrhoea among female children.

After adjustment for covariates, emotional violence (aPR: 0.32; 95% CI: 0.15–0.70) was associated with 68% lower prevalence of receiving treatment (Table 4). Sex-stratified analysis revealed that among female children, emotional violence (aPR: 0.09; 95% CI: 0.02–0.35) showed an 91% lower prevalence of receiving treatment (Table 4).

**Table 4:** Adjusted PR and 95% CI of the association between maternal exposure to different forms of IPV and child’s diarrhoea treatment, stratified by the sex of the child

Violence Variable	aPR^1^ (95% CI) Overall (*N* = 177)	aPR^2^ (95% CI) Male (*N* = 99)	aPR^2^ (95% CI) Female (*N* = 78)
Physical	0.84 (0.52–1.37)	1.00 (0.51–1.94)	0.98 (0.41–2.34)
Sexual	0.52 (0.23–1.20)	0.53 (0.15–1.83)	0.44 (0.11–1.76)
Emotional	**0.32 (0.15–0.70)**	0.56 (0.22–1.42)	**0.09 (0.02–0.35)**
Controlling behaviour	0.74 (0.51–1.08)	0.93 (0.54–1.61)	0.59 (0.31–1.12)
Any violence	0.71 (0.50–1.03)	0.86 (0.50–1.49)	0.65 (0.36–1.15)

aPR^1^ = Prevalence Ratio adjusted for covariates including region, residence, religion, household wealth index, partner’s education level, mother’s age, mother's education level, mother's occupation, number of children under five years in the household, child’s birth order number, child’s age in months, and child’s sex

aPR^2^ = Prevalence Ratio adjusted for covariates including region, residence, religion, household wealth index, partner’s education level, mother’s age, mother's education level, mother's occupation, number of children under five years in the household, child’s birth order number, and child’s age in months

*CI* Confidence Interva;

## Discussion

Our study found that 8.9% of children in the sample experienced diarrhoea, and just over half of these cases (56.6%) received treatment. Maternal exposure to various forms of IPV, particularly sexual violence, emotional violence, and controlling behaviours, was significantly associated with an increased prevalence of child diarrhoea. Sex-stratified analysis indicated that these associations were more pronounced among male children. In contrast, maternal exposure to emotional violence was significantly associated with lower prevalence of seeking treatment for child diarrhoea. This association appeared stronger among female children.

The association between maternal exposure to IPV and child diarrhoea outcomes in this study are consistent with a growing body of evidence from other LMICs. A large-scale analysis using DHS datasets from 37 sub-Saharan African countries similarly reported increased odds of childhood diarrhoea among children whose mothers experienced physical, sexual, or emotional violence [7]. In Cambodia, an analysis of DHS data spanning over a decade found higher odds of diarrhoea among children of women who had experienced any type of IPV [9]. Within the South Asian region, a study pooling survey data from Nepal, Bangladesh, and India found that physical and/or sexual violence significantly increased the likelihood of child diarrhoea [25]. The association between maternal exposure to IPV and child diarrhoea outcomes may be due to poorer physical and mental health of the exposed mothers resulting in reduced care of the children [8, 25]. Additionally, witnessing IPV increases children’s psychological stress which in turns increases their risk of illness [46].

While previous studies found a significant relationship between maternal exposure to physical violence and child diarrhoea, this study did not find the same results. Additionally, none of these studies looked at maternal exposure to controlling behaviours which this study found to be significantly associated with child diarrhoea, suggesting the need for broader conceptualisations of IPV in relation to child health.

The current findings also align with previous research that links IPV with reduced health-seeking behaviour. In the Cambodian DHS study [9], fewer children whose mothers had experienced IPV received treatment for diarrhoea compared to those whose mothers had not (32.0% vs. 39.5%), consistent with this study’s finding that emotional violence were associated with significantly lower treatment-seeking for childhood diarrhoea. This relationship may be mediated by reduced maternal autonomy, psychological distress, or disrupted care-seeking pathways, as suggested in prior literature [15]. This study strengthens the argument by showing that the negative association of IPV on treatment-seeking persists even after adjusting for socioeconomic and demographic confounders.

While this study did not find significant child sex differences in overall diarrhoea prevalence or treatment-seeking within the study sample, notable differences emerged in the sex-stratified analysis of IPV exposure. Specifically, IPV was more strongly associated with diarrhoea prevalence among male children and with reduced treatment-seeking among female children. Studies using the Gambian DHS data showed that male children had higher odds of diarrhoea occurrence, while female children had lower odds of receiving treatment for diarrhoea [47, 48]. This may be explained by a higher susceptibility of childhood infections in male children compared to females. Additionally, boys may have more freedom to leave their homes and acquire infections than girls [49]. Broader studies across South Asia also show that care-seeking behaviours are often shaped by gender norms, with girl children frequently disadvantaged in terms of access to healthcare services [50]. The unique contribution of this study is its focus on how IPV modifies these gendered patterns.

Strengths and Limitations.

This study has several important strengths. First, the use of nationally representative data enhances the generalisability of the results to the broader population of Nepal. Second, the analysis accounted for the complex sampling design of the DHS, including both national-level clustering and clustering of children within mothers. Lastly, another strength is the completeness of the dataset. 

Nonetheless, several limitations must be acknowledged. As with all cross-sectional studies, causality cannot be inferred from these findings. The associations observed may reflect correlation rather than a direct effect of violence on child health outcomes. Additionally, self-reported data, especially regarding sensitive issues such as IPV, are subject to reporting bias. While this study aimed to comprehensively include all covariates that may influence the multivariate analysis, some variables such as water, sanitation and hygiene conditions may be missed due to lack of data standardization. Finally, while the overall sample size was robust, the number of children with diarrhoea for whom treatment-seeking behaviour could be analysed was relatively small (*n* = 177).

## Conclusions

Our findings revealed that exposure to sexual and emotional violence, as well as controlling behaviours, were significantly associated with increased child diarrhoea, with stronger effects seen in male children. Additionally, maternal exposure to emotional violence was linked to reduced treatment-seeking for diarrhoea, particularly among female children.

These findings have important implications for global health, particularly in countries where both child morbidity and woman’s exposure to IPV remain critical public health concerns. The consistent association between maternal exposure to violence and child diarrhoea underscores the need to adopt a holistic, multisectoral approach to child health which includes violence prevention and support for mothers.

At a policy level, integrating IPV screening and support services within maternal and child health programmes could help identify at-risk families and offer timely interventions. There should be a larger focus on often overlooked IPV including emotional violence and controlling behaviours, especially given their high prevalence. Moreover, addressing structural drivers of violence such as poverty, gender inequality, and lack of legal protection may have downstream benefits for child health. The gendered differences in treatment-seeking also highlight the need for gender-sensitive child health policies. Programmes should ensure that both male and female children have equal access to timely and appropriate healthcare.

## Data Availability

The data that support the findings of this study are available from The DHS Program but restrictions apply to the availability of these data, which were used under license for the current study, and so are not publicly available. Data are however available from the authors upon reasonable request and with permission of The DHS Program.

## Abbreviations

aPR: adjusted Prevalence Ratio
CEDV: Children’s Exposure to Domestic Violence
CI: Confidence Interval
DHS: Demographic and Health Survey
IPV: Intimate Partner Violence
LMICs: Low- and middle-income countries
SDG: Sustainable Development Goals
VAWG: Violence Against Women and Girls
WHO: World Health Organization
